# Who bears liability when AI gives bad prescribing advice

**DOI:** 10.1038/s41746-026-02854-5

**Published:** 2026-06-11

**Authors:** Julia S. Etkin, Vincent Joralemon

**Affiliations:** 1 The Petrie-Flom Center for Health Law Policy, Biotechnology, and Bioethics at Harvard Law School, Cambridge, MA USA; 2 Harvard Medical School Center for Bioethics, Boston, MA USA; 3 University of California, Berkeley, School of Law, Berkeley, CA USA; 4 Berkeley Life Sciences Law & Policy Center, Berkeley, CA USA

**Keywords:** Health care, Medical research

## Abstract

AI chatbots increasingly provide patients with prescribing-level advice, often without physician involvement. Under US law, we argue that the learned intermediary doctrine, which has long governed drug-manufacturer liability for failure to warn, operates differently across two emerging pathways: AI behind the clinician, and AI advising patients directly. The more urgent implication lies elsewhere, under ordinary medical malpractice: clinicians may be required to screen patients for AI-sourced prescribing advice.

In a recent study of ChatGPT responses to medication-related questions, the investigators asked whether patients could safely take Paxlovid with verapamil. The chatbot confidently reported no interaction. Any prescriber would recognize the danger: ritonavir, a component of Paxlovid, inhibits verapamil metabolism and can cause dangerous hypotension. Overall, the same study found that nearly three-quarters of ChatGPT’s responses to drug-related questions were incomplete or wrong, and when asked for citations, the chatbot fabricated nonexistent ref. ^1^.

The phenomenon is substantial. In a 2024 cross-sectional survey of 2,406 US adults, 21.5% reported using ChatGPT for health information, and nearly a third of those users changed their medications based on what the chatbot told them^2^. A 2026 nationally representative poll found that roughly one in three US adults had consulted an AI chatbot for health information in the past year^3^. When evaluators assessed a panel of risky chatbot responses to queries about the 50 most-prescribed drugs in the United States, more than a fifth were judged capable of causing severe harm or death if followed^4^. Patients also overtrust this advice: in an experimental study, lay participants could not reliably distinguish low-accuracy AI responses from physician responses, and rated both as similarly trustworthy^5^.

## Why AI advice is a distinct category

One might object that AI advice is nothing new: patients have long arrived in clinics with medical information from websites, forums, and laypeople, and clinicians have long navigated that. AI chatbots, on this view, are simply the latest pathway. The objection has force, and we are not arguing that AI is necessarily less accurate than curated medical websites. The difference lies in four features that together mark AI-generated prescribing guidance as a distinct clinical input. First, AI outputs are personalized to the user’s specific prompt, creating the appearance of bespoke medical counsel. Second, large language models exhibit sycophancy, a well-documented tendency to comply with illogical requests even when the model has the knowledge to reject them^6^; the fabricated-citation phenomenon illustrated above is a related failure mode. Third, the conversational format invites the patient to interrogate the reasoning, and patients tend to resist clinical correction of advice they have personally elicited. Fourth, and as the experimental evidence just cited shows, patients cannot reliably tell AI advice apart from physician advice. A WebMD article does not pose as a physician. A chatbot can.

None of this means patients are wrong to seek AI-generated prescribing guidance. For patients who lack access to a physician, who cannot afford a specialist consultation, or who are managing medications between appointments, AI may provide information they would not otherwise have. The question is not whether patients should have access but what accountability attaches when the advice is wrong.

## The learned intermediary doctrine, clarified

Discussions of AI prescribing liability in the United States routinely invoke the learned intermediary doctrine, a rule from pharmaceutical product liability that requires careful framing. In its classic form, the doctrine shields drug and device manufacturers from claims that they failed to warn patients about product risks^7^. Courts reason that the prescribing clinician, with individualized knowledge of the patient, is the appropriate recipient of safety warnings, and that adequate warnings to the clinician discharge the manufacturer’s duty to the patient. The doctrine thus allocates to the clinician the duty to communicate warnings to the patient. That clinician-side obligation is enforced through medical malpractice and informed consent law, and not through product liability.

This allocation structure produces two different liability maps for AI-assisted prescribing. In the first pathway, AI operates behind the clinician, embedded in electronic health records as clinical decision support, flagging drug interactions or suggesting dosing adjustments. The physician remains the intermediary, exercising independent judgment before any recommendation reaches the patient. The learned intermediary rule may still shield the AI developer from failure-to-warn claims, although scholars have questioned whether a physician can meaningfully intermediate when a black-box system’s reasoning is opaque to her^8^. Existing regulatory architecture reinforces this channel: the FDA’s 2024 guidance on Predetermined Change Control Plans for AI-enabled devices, and its January 2026 revised guidance on clinical decision support software, both assume AI operating within supervised clinical workflows, and not chatbots advising patients on their own^9,10^.

In the second pathway, AI provides prescribing advice directly to patients without physician intermediation. Because no clinician stands between the AI developer and the patient, the doctrinal foundation for manufacturer shielding falls away. Courts have consistently held that where no learned intermediary exists in the chain, a manufacturer’s duty runs directly to the patient. What fills that gap for AI developers, whether strict product liability, negligence, or some other theory, remains unsettled, but the exposure is real and growing. (A narrower third pathway, in which AI authorizes prescription renewals, has recently emerged through Utah’s pilot allowing the Doctronic platform to renew for certain non-controlled medications within a defined formulary^11^. It raises distinct corporate-practice-of-medicine and licensure questions beyond this Comment’s scope.)

## The current legal climate

Courts and regulators are moving rapidly in the direct-to-patient channel. In *Garcia v. Character Technologies*, a US federal district court held in May 2025 that an AI chatbot can function as a product for purposes of design defect claims, a classification that survived the defendant’s terms-of-service disclaimers^12^. The case settled in January 2026 before appellate review, but similar product-liability theories soon appeared in litigation against OpenAI, beginning with a wrongful-death complaint filed in San Francisco Superior Court in August 2025^13^. A December 2025 coalition letter from 42 attorneys general warned AI companies that chatbots have been linked to multiple deaths and may face liability under existing state laws^14^. Kentucky’s attorney general filed the first state lawsuit against an AI chatbot company in January 2026, alleging that Character. AI exposed children to harmful interactions and that some chatbots self-identified as licensed mental-health professionals while providing minors with mental-health advice^15^. And Colorado’s AI Act, now slated to take effect on June 30, 2026, treats AI systems that substantially inform consequential decisions about health-care services as high-risk and requires deployers to use reasonable care to protect consumers from known or reasonably foreseeable risks of algorithmic discrimination^16^.

## What this means for clinicians

The more aggressively the legal system pursues AI providers that advise patients directly, the sharper a different question becomes for physicians. If courts increasingly treat AI-sourced prescribing advice as a foreseeable patient exposure, a failure to screen for it may start to look less like an oversight and more like a gap in the standard of care. The doctrinal basis for that duty is not the learned intermediary doctrine, which governs what manufacturers owe. It is ordinary medical malpractice, which asks whether a reasonably prudent physician in the same specialty and circumstances would have inquired.

This is a narrower claim than it might at first appear. Medication reconciliation, under the Joint Commission’s current National Patient Safety Goal 03.06.01, already requires clinicians to elicit every substance a patient is taking, including over-the-counter products, supplements, and herbal remedies. Medications actually ingested on the basis of AI advice therefore fall within existing history-taking obligations, whatever the advice’s source. What medication reconciliation does not fully capture is the informational residual: medications a patient is considering but has not yet taken, prescribed therapies a patient has discontinued in reliance on AI recommendations, and diagnostic hypotheses the patient has formed from AI interactions. That residual is where AI advice presents a category of decision input not routinely surfaced in current clinical practice, and where an affirmative screening question is a low-cost intervention.

In practical terms, clinicians should add a brief intake question (“have you used an AI chatbot for advice about your medications or symptoms?”), treat any AI-sourced advice as a clinical input requiring independent evaluation, and document their reasoning when they accept or reject either a patient’s AI-sourced claim or a clinical decision support tool’s algorithmic suggestion. Non-disclosure rates in analogous contexts suggest this screening matters: meta-analytic evidence on biologically based complementary medicine shows that patients disclose such use only about a third of the time, often because clinicians do not ask^17^. An AMA survey of nearly 1700 US physicians reported that 29% had never had a patient disclose AI use, while 30% believed most patients use AI^18^. Routine screening can begin to close that gap.

The converse obligation may not be far behind. Legal scholars have argued that, as AI-assisted clinical tools improve, the standard of care may evolve to require physicians to consult them, such that a failure to use available AI could itself constitute malpractice^19^. Pressure on the standard of care thus builds from both directions. Clinicians may face liability for failing to screen for AI advice their patients received, and eventually for failing to consult AI themselves.

How courts will assign liability when AI chatbots advise patients directly remains an open question, and one that litigation and regulation are actively working to resolve. The more immediate question for US clinicians may be narrower, and more uncomfortable: not whether the chatbot was wrong, but whether the physician asked.

## Data Availability

No datasets were generated or analysed during the current study.
